# An RNF4-Based Tool for Tracking Subcellular Localization of PolySUMOylation During Cellular Stress

**DOI:** 10.3390/biom16050748

**Published:** 2026-05-20

**Authors:** Joseph S. Floramo, Yaguang Zhao, Lorna Cohen, Kristin Gallik, David Brass, Tao Yang

**Affiliations:** 1 Department of Cell Biology, Van Andel Institute, Grand Rapids, MI 49503, USA; jsfloramo@yahoo.com (J.S.F.);; 2 Optical Imaging Core, Van Andel Institute, Grand Rapids, MI 49503, USA; 3 Research Development, Van Andel Institute, Grand Rapids, MI 49503, USA

**Keywords:** polySUMOylation, cellular stress, nuclear puncta, microscopy

## Abstract

SUMOylation is a rapid and dynamic process that orchestrates the switch between complex assembly and disassembly and between protein stabilization and turnover, making it particularly suitable for regulating stress responses. While proteomic methodologies exist for analyzing SUMOylated proteins under stress conditions, methods/tools for visualizing polySUMOylation dynamics have not been established. Here, we develop a polySUMOylation tracking tool by fluorescently labeling the polySIM domains derived from RNF4, which can reliably track polySUMO location and relate polySUMOylation levels to puncta number and intensity under various stress conditions, such as serum starvation, oxidative stress, and genotoxic stress. Furthermore, we extend its utility for tracking polySUMOylation across multiple cellular contexts in both control and stressed states. Collectively, this tracking tool enables deeper investigation of polySUMOylation dynamics and advances our understanding of how polySUMOylation regulates cellular processes in stress responses and disease pathogenesis.

## 1. Introduction

SUMOylation, the covalent attachment of small ubiquitin-like modifier (SUMO) proteins to target substrates, is a crucial post-translational modification (PTM) that regulates diverse cellular processes [1]. In *Saccharomyces cerevisiae*, SUMOylation is carried out by a single isoform encoded by SMT3, whereas mammalian cells express four isoforms—SUMO1, SUMO2, SUMO3, and SUMO4. SUMO2 and SUMO3 share approximately 97% sequence identity, in contrast to SUMO1 (~50% identity), while SUMO4 is generally considered non-conjugatable under physiological conditions [2]. SUMO conjugation proceeds through an enzymatic cascade involving E1-activating, E2-conjugating, and E3-ligating enzymes, resulting in monoSUMOylation, multiSUMOylation, or polySUMO chain formation [3]. SUMOylation interacts with SUMO-interacting motifs (SIMs) to regulate protein complex assembly, stability, and turnover [4]. Whereas monoSUMOylation often promotes the assembly of multi-protein complexes, polySUMOylation frequently destabilizes substrates by recruiting SUMO-targeted ubiquitin ligases (STUbLs), leading to ubiquitination and complex disassembly [5]. Through these versatile functions, SUMOylation acts as a dynamic regulator of cellular responses to environmental and physiological stress.

As a reversible PTM, SUMOylation is tightly controlled by sentrin-specific proteases (SENPs), which differ in tissue distribution, subcellular localization, substrate preference, and susceptibility to stress [6]. SENP1–3 and SENP5 predominantly remove mono- and multiSUMOylation, whereas SENP6 and SENP7 preferentially edit or dismantle polySUMO chains [7]. Cells—particularly long-lived, postmitotic cell types—are continually exposed to proteotoxic and genotoxic stressors that accumulate over time and contribute to senescence, tissue degeneration, and age-related diseases. Altered SUMOylation patterns, often triggered by imbalances in SUMOylation or deSUMOylation enzyme activity, are commonly observed during aging or during exposure to experimental stress including heat shock, oxidative stress (e.g., hydrogen peroxide), and DNA damage [2,3].

Stress granules (SGs), dynamic assemblies of stalled translation-associated messenger ribonucleoproteins, are key structures formed during cellular stress [8]. Within PML nuclear bodies, SUMO–SIM interactions help promote SG biogenesis [5]. PolySUMOylation contributes to stress resolution by marking SG components for turnover; for example, the STUbL RNF4 recognizes polySUMO chains on SG proteins and ubiquitinates them to facilitate proteasomal degradation. Impairment of this pathway delays SG clearance and compromises recovery from stress [9]. These findings highlight the importance of SUMO-mediated regulation in coordinating stress response, adaptation, and cellular homeostasis.

Stress responses also contribute broadly to the pathogenesis of chronic diseases such as osteoarthritis (OA) [10]. SUMOylation regulates inflammatory pathways that drive stress granule formation in OA [5,11,12,13,14]. We previously reported that SENP6 deficiency leads to accumulation of SUMO-modified TRIM28, resulting in enhanced p53 signaling and elevated senescence-associated secretory phenotypes (SASPs) [11,15]. Moreover, several deSUMOylases (SENP1, SENP2, SENP3, and SENP7) are susceptible to heat- or oxidative stress-induced inactivation [16]. Many stress pathways—including metabolic and inflammatory signaling—intersect and reinforce one another, often converging on ROS-mediated inflammatory hubs [10]. Notably, many of these pathways are SUMO-regulated [12,17], underscoring the importance of understanding SUMO dynamics under stress conditions.

Despite extensive progress, tools enabling real-time visualization of SUMOylation in live cells remain limited. Although newly developed antibodies can detect SUMO-modified proteins, issues of isoform specificity persist. For example, while SUMO2 8A2 antibodies show high selectivity, many SUMO4 antibodies cross-react with SUMO2/3, and several SUMO2/3 antibodies also recognize SUMO4 [18]. Fluorescent probes such as KmUTAG, derived from the Ulp1 UD domain of *Kluyveromyces marxianus*, have advanced SUMO imaging but primarily detect individual SUMO moieties and do not distinguish monoSUMOylation from polySUMO chain formation [19]. Currently, no effective methodology exists to discriminate polySUMOylation from mono- or multiSUMOylation in living cells.

Here, we address this critical gap by developing a fluorescent system based on polySUMO-binding SIM arrays derived from RNF4. We demonstrate that fluorescently tagged polySIM domains reliably visualize polySUMO localization and allow for relative quantification of polySUMOylation dynamics through puncta number and fluorescence intensity under basal and stress conditions. This tool provides a powerful approach for dissecting SUMO signaling during stress responses and may offer new insights into the mechanisms linking SUMOylation to disease.

## 2. Materials and Methods

### 2.1. Cell Culture

HEK293T cells (ATCC, Manassas, VA, USA) were maintained in high-glucose DMEM (H-DMEM) (Gibco/ThermoFisher Scientific, Grand Island, NY, USA) supplemented with 10% fetal bovine serum (FBS)(Corning Inc, Corning, NY, USA) and 1% penicillin/streptomycin (Pen/Strep) (Gibco/ThermoFisher Scientific, Grand Isalnd, NY, USA). Cells were subcultured at a 1:6 ratio every 2–3 days. Stable cell lines expressing either wild-type (WT) or mutant (MT) RNF4 polySIM domains were maintained at ~70% confluence on 10-cm dishes. For imaging experiments, cells were seeded at ~30% confluence to allow stress treatments to begin upon reaching ~50% confluence on poly-D-lysine-coated ibidi 8-well microscopy slides (ibidi USA Inc., Fitchburg, WI, USA). ATDC5 cells (ATCC, Manassas, VA, USA) were cultured in α-MEM supplemented with 10% FBS and 1% Pen/Strep and subcultured at a 1:6 ratio every 4 days, with fresh media changes every 2 days.

### 2.2. Plasmids and Transfection

WT and MT RNF4 polySIM domain plasmids were kindly provided by the R. T. Hay laboratory (University of Dundee). Inserts were cloned into pCMV-eGFP-C3 vectors using a Gibson Assembly kit (Lot#E5510S, New England Biolabs, Inc., Ipswich, MA, USA). For viral production, GFP-tagged WT or MT RNF4 polySIM domain sequences were cloned into the pENTR3c backbone and transferred into a pLenti-CMV backbone via recombination.

HEK293T cells were transfected with pLenti-CMV constructs (10 µg), pMD2.G (5 µg), and psPAX2 (5 µg). Viral supernatant was collected 48 h post-transfection and cleared of cellular debris. pCMV-SENP6 and pCMV-HA-SUMO2 plasmids were generated previously. Transfections were performed using a 2 µg DNA to 6 µL PEI (1 mg/mL) ratio in Opti-MEM. Cells were allowed to express plasmids for 1–3 days prior to downstream applications.

### 2.3. Viral Transduction

Target cells were transduced using viral supernatant diluted 1:25 for HEK293T cells or 1:5 for ATDC5 cells, supplemented with polybrene (8 µg/mL) and CaCl_2_ (from a 1000× stock). Following transduction, cells were selected with blasticidin (6 µg/mL) and clonal populations were generated via serial dilution.

### 2.4. Stress Treatments

Serum Starvation: H-DMEM containing 1% Pen/Strep and no serum was applied to cells for 24 h at 37 °C and 5% CO_2_.

Arsenic Trioxide: A 1 mM As_2_O_3_ stock solution was diluted to a final concentration of 1 µM in H-DMEM complete media and applied to cells for 2 h. Fresh H-DMEM complete media was used for recovery when indicated.

Hydrogen Peroxide (H_2_O_2_): A 19.7 M H_2_O_2_ stock was serially diluted to final concentrations of 100 nM or 500 nM in H-DMEM complete media. Cells were treated for 5 or 10 min, respectively.

Doxorubicin: A 5 mM doxorubicin stock solution in DMSO was diluted to 5 µM in H-DMEM complete media and applied to cells for 4 h at 37 °C and 5% CO_2_.

45 °C Heat Shock: Cells were placed in a 45°C incubator with 5% CO_2_ for 45 min.

Following treatments, cells were fixed with freshly prepared 4% formalin, stained with N-cadherin antibody fo r 1 h, followed by Alexa Fluor 568 goat anti-rabbit secondary antibody for 1 h, and counterstained with DAPI.

### 2.5. Microscopy

Confocal z-stacks were acquired using a Zeiss LSM 880 confocal microscope equipped with an Axio Observer 7 inverted stand (Carl Zeiss Microscopy, LLC, White Plains, NY, USA) and controlled with Zen Black software (v2.3). Imaging was performed using 405 nm (diode), 488 nm (argon ion), and 561 nm (DPSS) laser lines, along with transmitted white light. Emission was collected through a Zeiss Plan-Apochromat 40×/1.2 NA water-immersion objective with a 42 µm pinhole, using multi-alkali PMT detectors for fluorescence and a T-PMT detector for transmitted light.

Images were acquired sequentially as 16-bit images at 1024 × 1024 pixel resolution with 0.5 µm z-steps, a pixel dwell time of 0.51 µs, and an optical zoom of 1.0.

### 2.6. RNF4 Tracking Tool Image Analysis

Original CZI files were imported into ImageJ/Fiji (v1.54f or v2.15.1) [20] for preprocessing. Channels were split and average z-projections were generated using the Z-Project function and saved as TIFF files. Nuclei were segmented from DAPI projections using the StarDist plugin with the Versatile (fluorescent nuclei) model and default settings. Resulting label images were saved as TIFF files.

Average projections of the DAPI, RNF4 (GFP), and N-cadherin channels, along with StarDist-derived nuclear masks, were imported into CellProfiler (v4.2.8) [21] to segment cell boundaries, identify GFP puncta, and quantify puncta localization within nuclear and cytoplasmic compartments. Nuclear objects smaller than 1200 pixels^2^ were excluded from downstream analysis.

Image intensity was rescaled using RescaleIntensity. GFP and RFP channels were combined using ImageMath to improve cell boundary detection, followed by Gaussian filtering (σ = 1). Cell bodies were segmented using IdentifySecondaryObjects (Otsu thresholding with three classes, middle class as foreground, correction factor = 0.5). Cytoplasmic compartments were generated using IdentifyTertiaryObjects.

GFP puncta were enhanced using a Gaussian subtraction approach (σ = 300 background subtraction followed by σ = 1 smoothing). EnhanceOrSuppressFeatures was applied (feature size = 4; slow mode). Puncta were identified using IdentifyPrimaryObjects (diameter 1–10 pixels; two-class Otsu thresholding; lower bound = 0.05), with clumped objects separated by shape and intensity. Puncta outside cell boundaries were excluded. Remaining puncta were assigned to nuclear or cytoplasmic compartments.

Intensity measurements were extracted from puncta, whole-cell, cytoplasmic, and nuclear regions using the original GFP channel. All measurements were exported as CSV files for statistical analysis. Unless otherwise specified, default module settings were used.

### 2.7. Statistical Analysis

All boxplots represent mean ± SD. Images shown are representative of nine images collected across four biological replicates. Puncta number and fluorescence intensity were analyzed using two-way ANOVA with multiple comparisons (*p* < 0.05). Puncta localization was analyzed using an unpaired Mann–Whitney test (*p* < 0.05). All statistical analyses were performed using GraphPad Prism (v10.2.2).

## 3. Results

### 3.1. PolySUMOylation Is Predominantly Localized in the Nucleus Under Control Conditions

**Figure 1:**
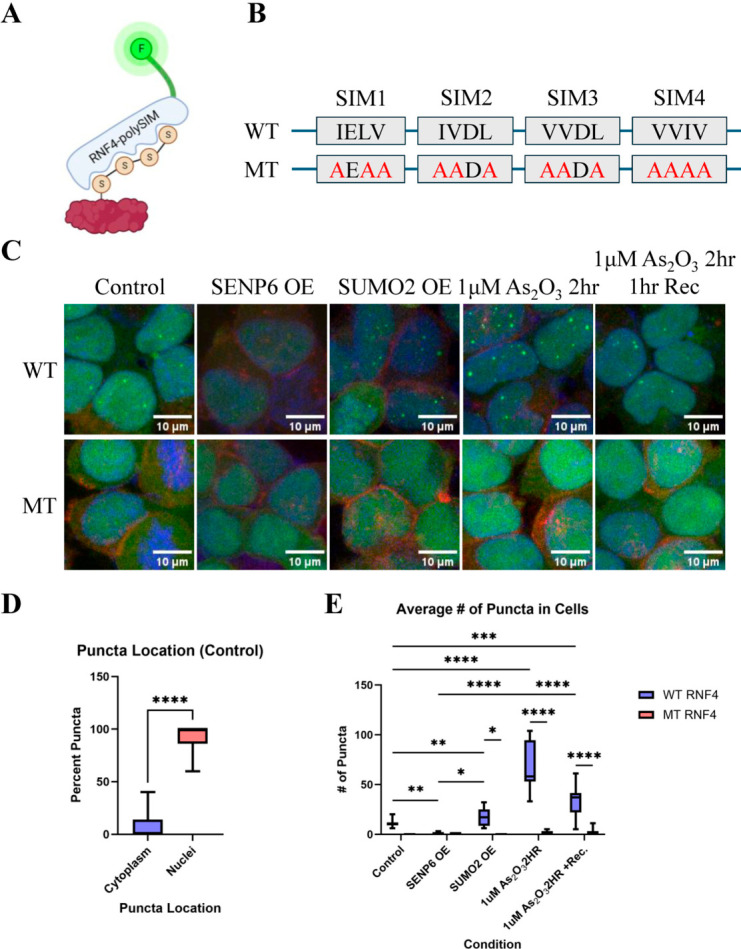
The RNF4 polySIM tracking tool is predominantly nuclear under basal conditions. (**A**) Schematic representation of the GFP-tagged RNF4 polySIM tracking construct and its interaction with polySUMOylated substrates. (**B**) Amino acid sequences of the wild-type (WT) and mutant (MT) RNF4 polySIM domains. (**C**) Representative fluorescence images of cells expressing the RNF4 polySIM tracking construct under the following conditions: control, SENP6 overexpression, SUMO2 overexpression, treatment with 1 μM As_2_O_3_ for 2 h, and 1 μM As_2_O_3_ for 2 h followed by a 1 h recovery period. The MT RNF4 polySIM construct was used as a negative control. Green, RNF4 polySIM; red, N-cadherin; blue, DAPI. (**D**) Quantification of the percentage of puncta localized to the cytoplasm versus the nucleus under control conditions (*n* = 9, **** *p* < 0.0001). (**E**) Quantification of puncta number per cell under the indicated conditions (*n* = 9, * *p* < 0.05, ** *p* < 0.01, *** *p* < 0.001, **** *p* < 0.0001).

To specifically visualize polySUMOylated proteins within cells, we developed a recombinant fluorescent polySUMO sensor using SUMO-interacting motifs (polySIMs) derived from RNF4. The polySIM domains of RNF4 were previously shown to preferentially bind polySUMO chains, with the highest affinity for tetra-SUMO2 chains, weaker affinity for tri-SUMO2 chains, and no detectable interaction with mono- or di-SUMO species [22]. These polySIM domains have also been used to identify polySUMO modifications in mass spectrometry applications, demonstrating their validated reliability compared to other less defined polySIM-containing proteins, such as Arkadia (RNF111), which also recognizes the SUMO1 cap [22,23,24,25,26]. Here, we fused the wild-type polySIM domains to GFP at the N-terminus and packaged it into a lentiviral vector (referred to as WT) to achieve stable, moderate expression in HEK293T cells (Figure 1A). To control for tracking specificity, as described by Tatham et al., each of the four RNF4 polySIM consensus sequences was mutated to alanine residues to abolish polySUMO binding (referred to as MT) [22] (Figure 1B). This mutant polySIM construct was similarly GFP-tagged and packaged into lentivirus.

Under control culture conditions, GFP-tagged WT polySIM domains were predominantly localized to the nucleus, appearing as distinct nuclear puncta (Figure 1C,D), whereas cells expressing the MT polySIM exhibited few to no detectable speckles (Figure 1C,E). We next assessed whether puncta formation correlated with changes in the cellular polySUMOylation state. Overexpression of SENP6, a deSUMOylase capable of removing or editing polySUMO chains, resulted in a marked reduction in the number of nuclear puncta (Figure 1C,E). Conversely, overexpression of SUMO2, which promotes polySUMO chain formation, led to an increase in nuclear puncta (Figure 1C,E).

We then examined whether stable expression of the recombinant polySIM domains interferes with endogenous RNF4 function. Endogenous RNF4 plays a key role in disassembling promyelocytic leukemia (PML) nuclear bodies, which contain highly mono- and polySUMOylated proteins [27]. Treatment of RNF4 polySIM–GFP-expressing cells with 1 µM As_2_O_3_ for 2 h resulted in a significant increase in the number of nuclear puncta (Figure 1C,E).

Following a 1 h recovery in fresh medium without As_2_O_3_, the number of puncta sharply decreased (Figure 1C,E), indicating that these structures can be efficiently cleared by endogenous RNF4.

Taken together, these results demonstrate that fluorescence-tagged RNF4 polySIM domains specifically recognize polySUMO chains and do not substantially interfere with endogenous RNF4 function, supporting their utility as a reliable tool for visualizing and tracking dynamic changes in polySUMOylation in cells.

### 3.2. Changes in PolySUMOylation Puncta Number and Localization upon Stressing Treatments

As SUMOylation pathways are frequently altered during cellular stress responses and their resolution, we next used the fluorescently tagged polySIM domains to track changes in puncta properties, including localization and number, in cells subjected to various stress conditions. These conditions included serum starvation, oxidative stress, induction of double-stranded DNA breaks, and heat shock. This approach enables monitoring of dynamic changes in polySUMO modifications across distinct cellular states.

**Figure 2:**
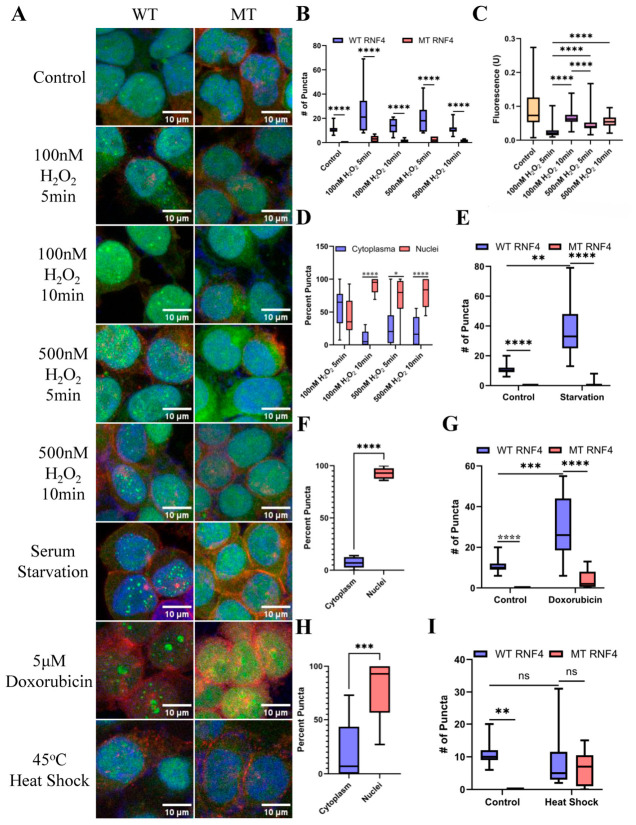
PolySUMOylation puncta number and subcellular localization change in response to cellular stress. (**A**) Representative fluorescence images of cells expressing the RNF4 polySIM tracking construct under the indicated stress conditions. Green, RNF4 polySIM; red, N-cadherin; blue, DAPI. (**B**) Quantification of puncta number in H_2_O_2_-treated cells (*n* = 9, **** *p* < 0.0001). (**C**) Quantification of the average fluorescence intensity of puncta in H_2_O_2_-treated cells (*n* = 48, **** *p* < 0.0001). (**D**) Quantification of puncta subcellular localization in H_2_O_2_-treated cells (*n* = 9, * *p* < 0.05, **** *p* < 0.0001). (**E**) Quantification of puncta number under serum starvation (*n* = 9, ** *p* < 0.01, **** *p* < 0.0001). (**F**) Quantification of puncta subcellular localization under serum starvation (*n* = 9, **** *p* < 0.0001). (**G**) Quantification of puncta number following 4 h treatment with 5 μM doxorubicin (*n* = 9, *** *p* < 0.001, **** *p* < 0.0001). (**H**) Quantification of puncta subcellular localization following 4 h treatment with 5 μM doxorubicin (*n* = 9, *** *p* < 0.001). (**I**) Quantification of puncta number in HEK293T cells subjected to heat shock at 45 °C (*n* = 9, ** *p <* 0.01).

We evaluated the relationship between oxidative stress intensity and the formation of polySUMO-containing puncta. HEK293T cells were treated with 100 nM or 500 nM H_2_O_2_ for 5 or 10 min under conditions that did not induce apoptosis. Compared to controls, treatment with 100 nM H_2_O_2_ for 5 min was sufficient to induce a significant increase in puncta number, although puncta number did not further increase at longer treatment durations or higher H_2_O_2_ concentrations (Figure 2A,B). Relative increases in fluorescence intensity were observed following a 10-min treatment with 100 nM H_2_O_2_, as well as after 5- and 10-min treatments with 500 nM H_2_O_2_, compared with the 5-min treatment condition (Figure 2A,C). Notably, all treatment groups exhibited reduced fluorescence intensity compared to control conditions. This reduction is likely attributable to oxyradical-induced GFP denaturation and fluorescence quenching [28]. Therefore, comparisons among treatment conditions provide a more appropriate basis for interpretation.

Notably, the 100 nM H_2_O_2_ 5-min treatment condition led to a significant shift in puncta localization from the nucleus to the cytoplasm (Figure 2D). Previous studies have shown that SUMOylation is predominantly nuclear and plays critical roles in nuclear processes [29]. However, it has also been reported that SENP7 targets cytoplasmic proteins during the oxidative stress response, resulting in the accumulation of polySUMOylated substrates in the cytoplasm [30]. The shift from partially cytoplasmic to predominantly nuclear puncta may indicate a transition in the glutathione redox response toward oxidative DNA damage-associated transcription factors. (Figure 2D) [31,32]. These findings suggest that the intensity and duration of oxidative stress activate distinct stress responses, promoting polySUMOylation of stress-associated structures in different cellular compartments. The differences in puncta composition and stress body properties under varying oxidative stress conditions warrant further investigation.

Next, we evaluated the formation of polySUMOylated stress granules under serum starvation conditions, which resulted in a marked increase in puncta number per cell. These puncta remained predominantly nuclear, with a modest shift toward the cytoplasm (Figure 2A,E,F). Serum starvation deprives cells of nutrients and serum-derived signaling ligands. The pronounced increase in puncta formation under these conditions suggests that polySUMOylation plays an important role in mitigating acute metabolic stress or facilitating adaptation to changes in the signaling environment. Consistent with this, it has been reported that under serum starvation, HEK293T cells undergo a metabolic shift toward increased lactate utilization to support protein production [33]. Further characterization of the composition and function of these polySUMOylated puncta under metabolic stress conditions is warranted.

PolySUMOylated proteins have previously been reported as a consequence of heat shock-induced stress [13]. To assess whether these changes could be tracked in our system, HEK293T cells were subjected to heat shock at 45 °C. Under these conditions, no change in puncta number was observed relative to control samples, and no difference was detected between WT and mutant conditions (Figure 2A,I). This may suggest that heat shock induces damage to the RNF4 polySIM domains, impairing their ability to bind polySUMOylated substrates. These findings differ from prior reports demonstrating successful detection of polySUMOylated proteins following heat shock, which may reflect methodological differences. In previous studies, RNF4 polySIM domains were used to capture polySUMO-modified proteins after heat shock, without being directly exposed to the stress condition themselves [13]. Together, these results suggest a potential limitation of using stably expressed GFP–RNF4 polySIM domains to monitor certain stress responses, which should be considered in downstream application.

Finally, to assess changes in polySUMOylation in response to genotoxic stress, cells were treated with 5 µM doxorubicin for 4 h. This treatment resulted in a significant increase in nuclear puncta (Figure 2A,G,H). SUMOylation is well documented to play an essential role in double-stranded DNA repair, including the recruitment of SUMO E3 ligases PIAS1 and PIAS4, which are required for homologous recombination and non-homologous end-joining pathways [34]. In addition, recruitment of the E3 ligase CBX4 together with Polycomb Repressor Complex 1 (PRC1) to sites of DNA damage has also been reported [35]. Consistent with these findings, our data shows that stable expression of the polySIM domain exhibits distinct changes in both the abundance and localization of polySUMO-associated speckles in response to genotoxic stress.

### 3.3. Validation of the PolySUMOylation Tracking Tool in Additional Cell Types

**Figure 3:**
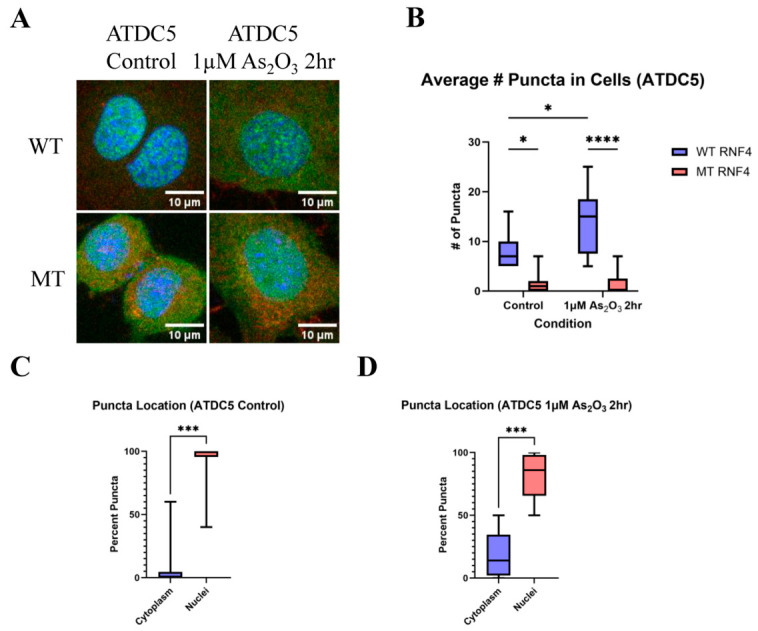
The RNF4 polySIM tracking tool functions in additional cellular contexts. (**A**) Representative fluorescence images of ATDC5 cells expressing the RNF4 polySIM tracking construct under control conditions or following treatment with 1 μM arsenic trioxide for 2 h. Green, RNF4 polySIM; red, N-cadherin; blue, DAPI. Scale bar, 40 μm. (**B**) Quantification of the average number of puncta per cell in ATDC5 cells (*n* = 9, * *p* < 0.05, **** *p* < 0.0001). (**C**) Quantification of puncta subcellular localization under control conditions (*n* = 9, *** *p* < 0.001). (**D**) Quantification of puncta subcellular localization following 2 h treatment with 1 μM As_2_O_3_ (*n* = 9, *** *p* < 0.001).

Given that polySUMOylation is a ubiquitous modification across cell types, we next generated an additional cell line stably expressing fluorescent polySIM domains. ATDC5 cells were used to validate control conditions observed in HEK293T cells. Under control conditions, the number of puncta was comparable between WT and mutant GFP-tagged RNF4 polySIM domain-expressing cells (Figure 1C,E and Figure 3A,B). To assess the response to oxidative stress, cells were treated with 1 µM As_2_O_3_ for 2 h to induce PML nuclear bodies. Consistent with observations in HEK293T cells, an increase in puncta number was detected (Figure 1C,E and Figure 3A,B). Under basal conditions, puncta were predominantly localized to the nucleus (Figure 1D and Figure 3C), and this nuclear localization was largely maintained following As_2_O_3_ treatment, albeit with greater variability (Figure 3D). These findings suggest potential cell type-specific differences in polySUMOylation dynamics under stress conditions. Overall, the results support the use of fluorescent RNF4 polySIM domains as a reliable tool for visualizing and tracking relative changes in polySUMOylation across different cell types.

## 4. Discussion

SUMOylation is a rapid and highly dynamic post-translational modification that enables reversible transitions between complex assembly and disassembly, as well as between protein stabilization and turnover, making it particularly well suited for regulating cellular stress responses. One of the best-characterized functions of polySUMOylation is its role in targeting modified proteins for degradation via SUMO-targeted ubiquitin ligases (STUbLs), thereby promoting the clearance of stress granules [5]. Although proteomic approaches have been developed to identify polySUMOylated proteins under stress conditions, tools for dynamic visualization of polySUMOylation in living cells have been lacking. To address this gap, we fluorescently labeled the polySIM domains of RNF4, enabling visualization of changes in polySUMOylation dynamics under both control and stress conditions through relative quantitative analysis of puncta number, intensity, and subcellular localization.

Under control conditions, RNF4 polySIM-labeled puncta were predominantly localized in the nucleus. The specificity and reliability of this tool were validated by the loss of puncta following SENP6 overexpression and the increase in puncta upon SUMO2 overexpression. Importantly, expression of the RNF4 polySIM tracking tool did not interfere with endogenous RNF4 function, as arsenic trioxide-treated cells exhibited normal puncta clearance during recovery. When cells were subjected to oxidative stress, serum starvation, or DNA damage, each condition resulted in increased puncta number and/or intensity, accompanied by distinct changes in subcellular localization. At 100 nM H_2_O_2_, where a slight shift toward cytoplasmic localization was observed, which may reflect modification of cytoplasmic redox pathways [36]. With prolonged exposure or at a higher concentration (500 nM), the puncta shift toward nuclear localization, suggesting potential regulation of oxidative stress-responsive transcription factors [37]. While other reported stressors predominantly induce nuclear puncta accumulation, these patterns likely represent stress-specific modifications of transcription factors or other regulatory proteins unique to each condition [14,32]. These localization patterns likely reflect the subcellular sites at which polySUMOylated substrates accumulate in response to specific stressors.

However, not all stress conditions were compatible with the RNF4 polySIM tracking tool. Following heat shock at 45 °C, few to no puncta were detected (Figure 2A,I). One possible explanation is the structural flexibility of the RNF4 polySIM domains, which lack rigid secondary structure and rely on biochemical affinity for substrate interaction. Heat-induced instability may therefore disrupt polySIM–polySUMO interactions under these conditions. Although recombinant RNF4 polySIM has been successfully used for affinity purification of polySUMOylated proteins from lysates of heat-shocked cells [23], in those experiments, the polySIM was added post-lysis and was not itself exposed to elevated temperatures. These observations indicate that the tracking tool has limitations under certain stress conditions and should be validated with appropriate controls. To broaden its applicability, future studies may explore alternative polySIM domains derived from more structurally stable polySUMO-binding proteins.

We further demonstrate that the RNF4 polySIM tracking tool can be applied across different cell types, including ATDC5 cells (Figure 3A–D), with results comparable to those observed in HEK293T cells. This finding suggests that the tool can be extended to investigate polySUMOylation dynamics in diverse cellular contexts and disease-relevant states. When applying this tool across cell fate transitions, potential transgene silencing during differentiation should be considered, as the current lentiviral delivery method results in random genomic integration that may be subject to epigenetic repression [38]. Targeted insertion of the tracking construct into genomic safe harbor loci using CRISPR–Cas–based approaches may provide a viable strategy to overcome this limitation.

Collectively, this polySIM-based tracking tool offers a new opportunity to study polySUMOylation dynamics. While this study focused on capturing and visualizing changes in fixed cells, the stable expression of this tracking tool enables its application to live cell imaging during stress treatment and recovery, enabling visualization of dynamic responses over time. This approach provides relative quantification of polySUMO chain abundance and, when integrated with established RNF4-based biochemical and proteomic methods for identifying polySUMOylated substrates, will offer a more comprehensive understanding of how polySUMOylation regulates cellular processes during stress responses and disease pathogenesis.

## 5. Conclusions

In this study, we developed a genetically encoded imaging tool based on RNF4-derived polySUMO-interacting motifs that enable specific visualization and relative quantitative analysis of polySUMOylation in cells. This approach overcomes a key limitation by distinguishing polySUMOylation from other SUMO modifications in situ. Using this system, we show that polySUMOylation is dynamically regulated under oxidative, metabolic, and genotoxic stress, with distinct changes in abundance and subcellular localization.

Importantly, this sensor reports polySUMOylation dynamics without disrupting endogenous RNF4 function and is applicable across multiple cell types. This tool provides a platform for investigating SUMO-dependent regulation of cellular organization and stress responses, and will facilitate future studies integrating biochemical and proteomic approaches to define polySUMOylation function in health and disease.

## Data Availability

The original contributions presented in this study are included in the article. Further inquiries can be directed to the corresponding author.
